# Sustainable Development of the Cement Industry and Blended Cements to Meet Ecological Challenges

**DOI:** 10.1100/tsw.2003.23

**Published:** 2003-05-05

**Authors:** Konstantin Sobolev

**Affiliations:** European University of Lefke, Civil Engineering Department, Lefke, Cyprus

**Keywords:** sustainability, cement, blended, ecology, development, technology, natural resources, industrial by-products, additive, energy consumption, strength, durability

## Abstract

The world production of cement has greatly increased in the past 10 years. This trend is the most significant factor affecting technological development and the updating of manufacturing facilities in the cement industry. Existing technology for the production of cement clinker is ecologically damaging; it consumes much energy and natural resources and also emits pollutants. A new approach to the production of blended or high-volume mineral additive (HVMA) cement helps to improve its ecological compatibility. HVMA cement technology is based on the intergrinding of portland cement clinker, gypsum, mineral additives, and a special complex admixture. This new method increases the compressive strength of ordinary cement, improves durability of the cement-based materials, and - at the same time - uses inexpensive natural mineral additives or industrial by-products. This improvement leads to a reduction of energy consumption per unit of the cement produced. Higher strength, better durability, reduction of pollution at the clinker production stage, and decrease of landfill area occupied by industrial by-products, all provide ecological advantages for HVMA cement.

## INTRODUCTION

According to Cembureau's report[1], the world production of cement has increased by about 50% in the past 10 years (Fig. 1). The growing demand for cement is the most significant factor affecting technological development and the updating of manufacturing facilities in the cement industry[2,3,4,5,6,7,8,9,10,11,12].

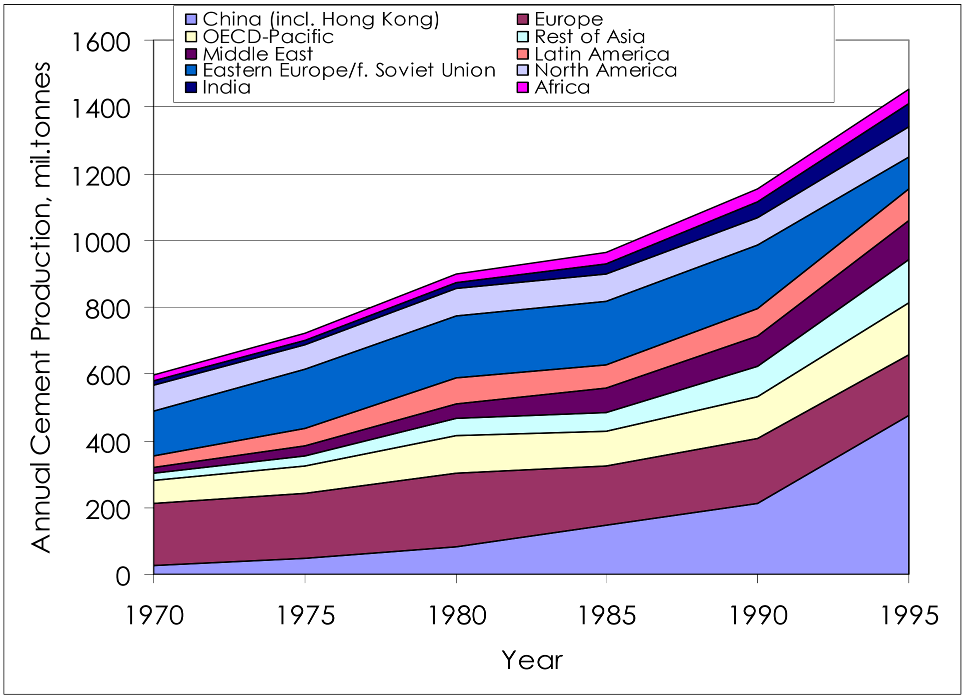

**FIGURE 1**. Production trends in the cement industry[1].

It is generally agreed that the production of cement is expensive and ecologically harmful. $CO_2$ (a principal gas contributing to the "greenhouse effect"), $NO_x$, and $SO_x$ are among the hazardous emissions generated in relatively high volumes by the conventional portland cement process.

If the production of cement is further expanded based on the existing technologies, products of the same performance will be obtained, but at a cost of increasing the consumption of raw materials and energy. Such expansion would also add a substantial degradation of the environment. As a result, the demand of growing markets cannot be met by simply extending the capacity of the current cement industry[11].

Sustainable development is the main way ahead. The environmental damage of construction materials can be measured and factored as an economic cost[13,14,15,16,17,18]. New products must either have better performance or use less raw materials and energy.

## SUSTAINABLE DEVELOPMENT AND ECO-EFFICIENCY

The definition of "sustainability", following the World Commission on Environment and Development, emphasizes the importance of ensuring the satisfaction of present need without compromising the ability of future generations to meet their own requirements[13]. As a business model, this means businesses try to balance inputs and outputs to maintain a profitable enterprise. Sustainable development also includes the issue of environmental impact, resource use, and social effects. Sustainable development can be defined as achieving social, economic, and environmental objectives in parallel[14,15]. For the construction industry sustainability means:

- Progress that meets the needs of society
- Economic development

- Preservation of the environment
- Efficient use of resources

The concept of eco-efficiency was formulated at the 1992 “Earth Summit”. The World Business Council for Sustainable Development (WBCSD) has proposed the following definition: “Eco-efficiency is reached by the delivery of competitively priced goods and services that satisfy human needs and bring quality of life, while progressively reducing ecological impacts and resource intensity throughout the life cycle, to a level at least in line with the earth’s estimated carrying capacity”[15,16,17,18]. WBCSD has identified seven elements to achieve eco-efficiency:

- Reduce the material intensity of products
- Reduce the energy intensity of products
- Reduce toxic emissions
- Enhance material recyclability
- Maximize sustainable use of renewable resources
- Extend product durability
- Increase the service life of the products

For the cement industry these components can be presented in the following guidelines[11]:

- Implementation of low-temperature technologies for manufacturing clinker (including belite and mineralized clinkers) with consequent energy/fuel savings
- Production of stronger cement and consequent application of stronger concrete in structural members with reduced cross-sections and consequent reduction of the amount of concrete raw materials, structural steel, and energy used, and reducing bulk consumption of cement and concrete
- Production of more durable cement-based materials with a significantly prolonged service life and reduced cost of maintenance
- Application of mineral additives or industrial by-products in blended cements and reducing energy consumption and the corresponding emissions per unit of cement produced
- Implementation of modern technologies for saving energy and recovery of materials leading to a reduction of heat, dust, and pollutant emissions
- Development and application of new types of cement based on alternative (different from conventional portland cement) binders

## THE INTEGRAL EFFICIENCY OF THE CONSTRUCTION MATERIALS

The formula for the integral efficiency of the construction material or structure can be presented in the following equation[18]:

$$\mathbf{L_i = \frac{\Sigma L_{1..n}}{\Sigma M_{1..n}} * D * W}$$

In this formula $\Sigma L_{1..n}$ represents the total engineering benefits as a sum of individual efficiency parameters $L_1$, $L_2$, .. $L_n$. $\Sigma M_{1..n}$ relates it to the sum of the expenditure $M_1$, $M_2$, .. $M_n$. The integral efficiency $L_i$ of the construction material increases with increasing of useful life D.

The fourth important efficiency variable is the reusability ratio W. This describes the proportion of the structure that can be appropriately reused after the end of the product's service life.

- Increase the engineering or ecological benefit
- Reduce expenditure
- Improve durability and service life
- Raise the recycling ratio

## TECHNOLOGICAL TRENDS IN CEMENT AND CONCRETE INDUSTRY

The most recent developments in cement and concrete technology can be summarized as follows[19]:

- <u>Strength:</u> Following a historical trend of cement and concrete development, there is a strong trend to increasing strength. Continuous breakthroughs in knowledge allow the design and application of super high-strength concrete with a compressive strength of 130–250 MPa, high flexural strength, and remarkable ductility[2,3,4,5,6,7,8,9,10,11,19,20,21,22]. As a result, research and application of effective structures, utilizing high-strength concrete, has been phenomenal in past years, demonstrating strong growth potential.
- <u>Chemical Admixtures:</u> The use of chemical admixtures has become one of the essential parts of modern concrete. Added to the concrete mixture, relatively small amounts of the chemical admixtures radically alter the behavior of fresh or hardened concrete. Modern admixtures provide a solution for overcoming or improving almost any problematic property of concrete[4,5,6,7,8,9,23].
- <u>Waste Utilization:</u> The utilization of industrial by-products and waste (IBPW) as mineral additives comprises a valuable segment of cement and concrete technology. According to wide-scale investigations[2,3,4,5,6,7,8,9,10,11,24,25,26,27,28,29,30,31,32,33,34,35,36,37, 38], the performance of concrete with controlled volumes of IBPW can be significantly improved. Well-investigated mineral additives include granulated blast furnace slag (GBFS), fly ash, and silica fume. These mineral additives not only yield concrete with improved properties and economical effectiveness, but they also improve the eco-balance of these materials[39].

As a result of these developments, the concept of high-performance concrete (HPC) has been put forward and successfully applied worldwide. It is generally accepted that concrete with properties better than conventional levels (considering its workability, strength, permeability, durability, etc.) could be called HPC. According to Forster[40] HPC is "a concrete made with appropriate materials combined according to a selected mix design and properly mixed, transported, placed, consolidated, and cured so that the resulting concrete will give excellent performance in the structure in which it will be exposed, and with the loads to which it will be subjected for its design life."

To realize the HPC concept, a variety of chemical admixtures and mineral additives are necessary, and a modern concrete batching plant must use adequate equipment for precise control, dispatching, dosing, and batch processing[19,23]. The technology of HPC was developed to simplify production and to extend its range of application[11].

## PROS AND CONS OF BLENDED CEMENT

The earliest reference to Roman cement and concrete of remarkable durability was made by Vitruvius (100 A.D.). The secret of the durability of Roman cement was to mix slaked lime with *pozzolana*, a volcanic ash from Mount Vesuvius. This resulted in a binder that was able to harden under water. Modern blended cements also utilize pozzolan as a mineral additive or cement-replacing material, which is intergrinded or blended with portland cement.

According to studies[26,41], pozzolans can be defined as "siliceous or siliceous -and-aluminous materials which by themselves possess little or no cementitious value but, in finely divided form and in the presence of moisture, chemically react with calcium hydroxide at ordinary temperatures to form compounds possessing cementitious properties," Pozzolans can be divided into two major groups[26]:

- Natural pozzolans (naturally occurring materials: volcanic ash/tuff, thermally activated clay/shale, diatomaceous earth) and
- Artificial pozzolans (including IBPW)

In spite of the evident savings from energy and raw materials there is a limit to the volumes of pozzolan utilized as cement/concrete additives. These volumes are controlled by the possible detrimental effect of pozzolanic additive on concrete performance (especially strength, permeability, durability). Among the major concerns of the misuse of mineral additives are reduced early strength, increased water demand of concrete mixtures, reduced freezing, and thawing resistance. The possible adverse effect of blended cements on the properties of concrete must be precisely evaluated; then the specific requirements of the particular construction project can be met.

## TECHNOLOGY OF HP/HVMA CEMENT: HIGH STRENGTH AND DURABILITY

A new reactive silica-based complex admixture was developed[11,24] to provide an improvement of cement properties. When added during the cement grinding process, the complex admixture modifies the surface and size distribution of cement particles; it also leads to the formation of highly reactive amorphous structures and prehydrates. The process named "high-performance cement technology" was found to be very effective for the development of an additional feature of normal cement. HPC can be defined as a product manufactured by the mechanochemical activation of certain proportions of clinker, gypsum, complex admixture, and, optionally, a mineral additive of industrial or natural origin, which imparts high strength and extreme durability to the concrete or mortar made from it[11]. The flowchart for the grinding unit for manufacturing HP/HVMA (high-volume mineral additive) cement is presented in Fig. 2.

The high-strength phenomenon was used to engineer a cement with HVMA. Because of its high strength, a large amount (up to 70%) of portland cement clinker could be replaced with mineral additives. A wide range of natural pozzolanic materials, sand, limestone, GBFS, fly ash, broken glass, and ceramic can all be used as mineral additives in these cements[24,42,43,44].

There are two types of HP cement:

1. Basic HPC (Type A)
2. Blended HPC (Type B)

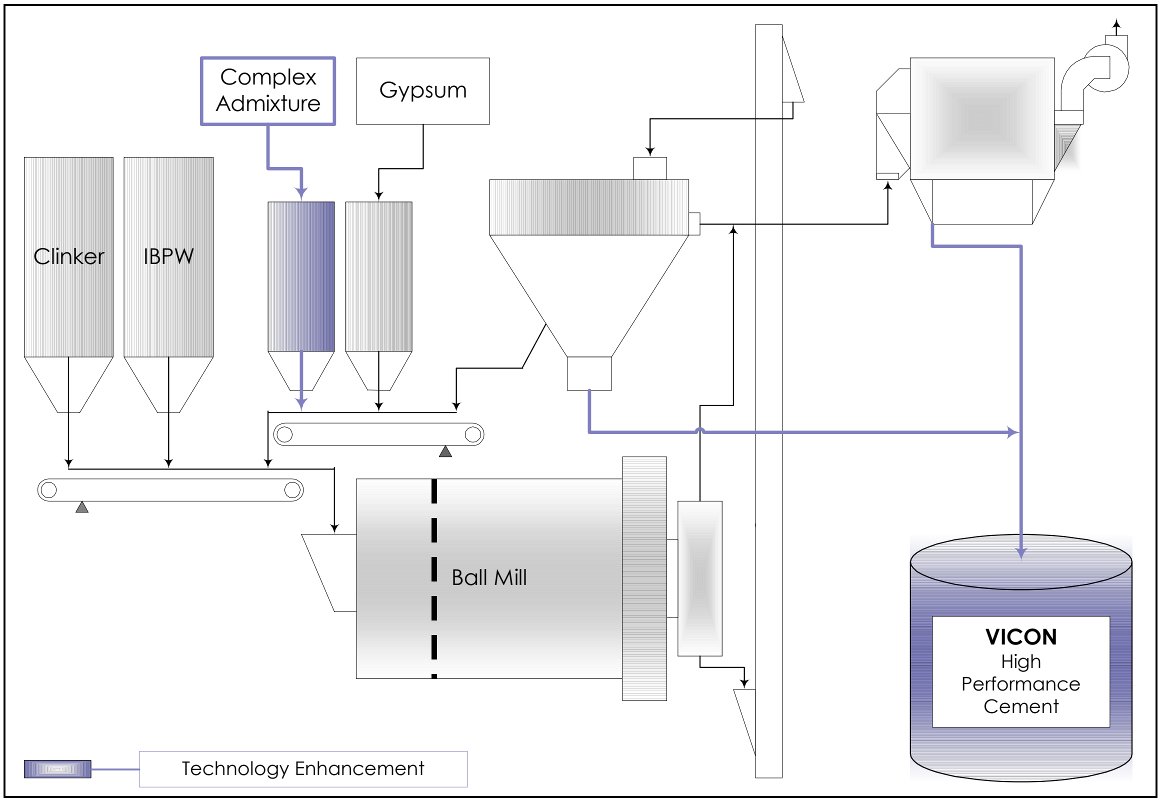

**FIGURE 2**. HPC technology.

Basic Type A cement involves intergrinding the clinker, gypsum, and a complex admixture. Blended Type B cement covers a wide range of HPCs with a mineral additive. The content of a mineral additive in blended HPC varies with the specified level of the properties and with the type of additive used. Wide-scale research[11,42,43,44] has demonstrated that HPCs with a mineral additive content within the standard limitations (25–50%) could be produced. Furthermore, the frames of mineral additive utilization in HVMA cement can be extended. New types of mineral admixtures (such as sand, broken glass, and ceramic) can be used as an ingredient in the blended HPC[24,44].

The chemical composition of HPC depends on the type and composition of the raw components. The chemical composition of HPC in comparison with normal portland cement (NPC) and GBFS is presented in Table 1.

**TABLE 1**
**Chemical Composition of HPCs[42]**

| | $SiO_2$ | $Al_2O_3$ | $Fe_2O_3$ | CaO | MgO | $K_2O$ | $Na_2O$ | $SO_3$ | L.O.I |
|---|---|---|---|---|---|---|---|---|---|
| NPC | 19.44 | 4.78 | 3.57 | 63.74 | 1.90 | 0.77 | 0.21 | 2.70 | 2.41 |
| GBFS | 40.09 | 11.48 | 1.56 | 31.85 | 9.49 | 1.00 | 0.00 | 2.10 | 0.17 |
| HPC-A | 28.47 | 4.18 | 3.36 | 55.01 | 1.89 | 1.02 | 0.38 | 2.61 | 2.83 |
| HPC-B | 34.28 | 7.83 | 2.46 | 43.43 | 5.69 | 1.01 | 0.19 | 2.35 | 1.50 |

The test results of HPC and NPC in accordance with the corresponding ASTM procedure are summarized in Table 2. HPC possesses a 28-day compressive strength, which is at least 65% higher than the strength of NPC. The early age strength development of the HPC classifies this cement as super rapid hardening cement. HPCs demonstrated an increase in a long-term strength, especially, in case of Type B based on 50% of GBFS (Fig. 3).

**TABLE 2**
**Physical Properties of HPC**

| | Fineness | | Setting Time (min) | | Normal Consistency % | Compressive Strength, MPa at age, days | | | | | |
|---|---|---|---|---|---|---|---|---|---|---|---|
| | Blaine ($m^2$/kg) | 45 µ | Initial | Final | | 1 | 2 | 3 | 7 | 28 | 90 |
| NPC | 310 | 8.50 | 165 | 205 | 27.1 | 26.2 | 36.4 | 42.4 | 48.5 | 57.1 | 64.7 |
| HPC-A | 570 | 5.40 | 100 | 145 | 18.5 | 44.3 | 55.9 | 62.2 | 74.1 | 94.4 | 96.2 |
| HPC-B | 580 | 5.20 | 175 | 225 | 17.5 | 35.2 | 44.8 | 54.2 | 65.6 | 92.7 | 105.5 |

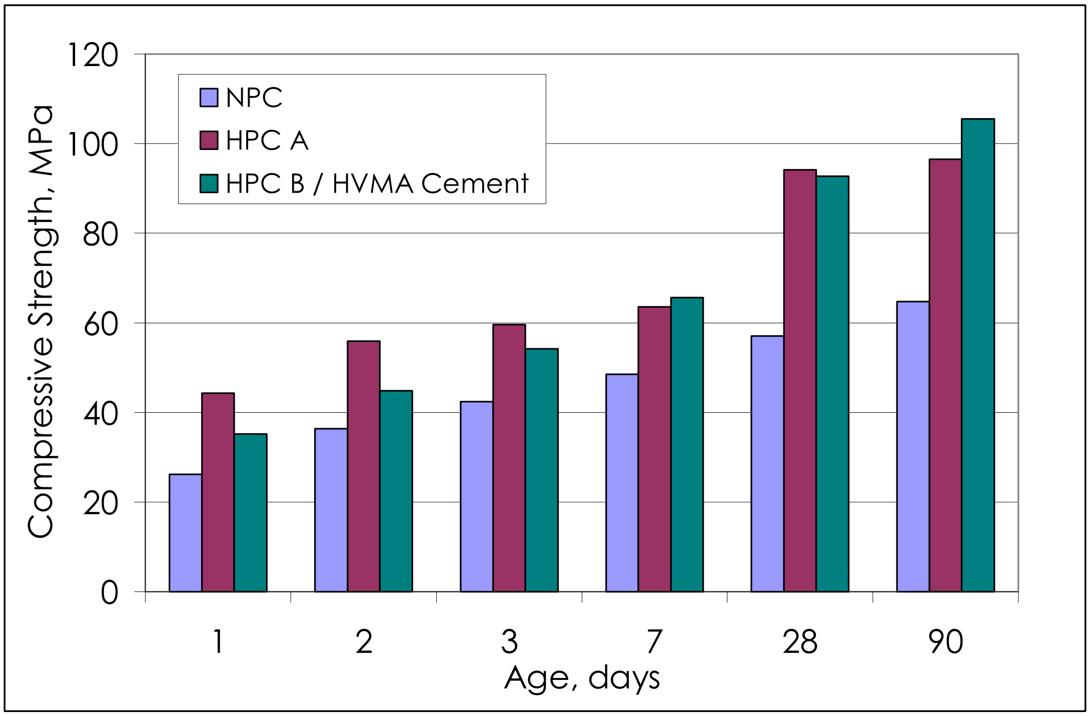

**FIGURE 3**. HP/HVMA cement strength development.

The application of GBFS in blended HPC provides a very high resistance to chemical attack[30,39,42]. Test results have demonstrated the possibility of producing blended HPC with a compressive strength of more than 80 MPa and a BFS content of up to 50% (Fig. 3).

As a result, HPC technology helps to overcome the problems related to the application of blended cements.

## IMPACT OF HP/HVMA CEMENT ON THE ENVIRONMENT

Blended cements incorporating different mineral admixtures or IBPW can partly replace the cement clinker. Clearly, blended cements meet the challenges of modern society by increasing bulk production and conserving energy[2,3,4,5,6,7,8,9,10,11,24,36,37]. In the case of $CO_2$ emissions "frozen" by the E.U. and U.S. at the level of 2000, the share of conventional portland cement in the market must be drastically reduced by the year 2015 (Fig. 4).

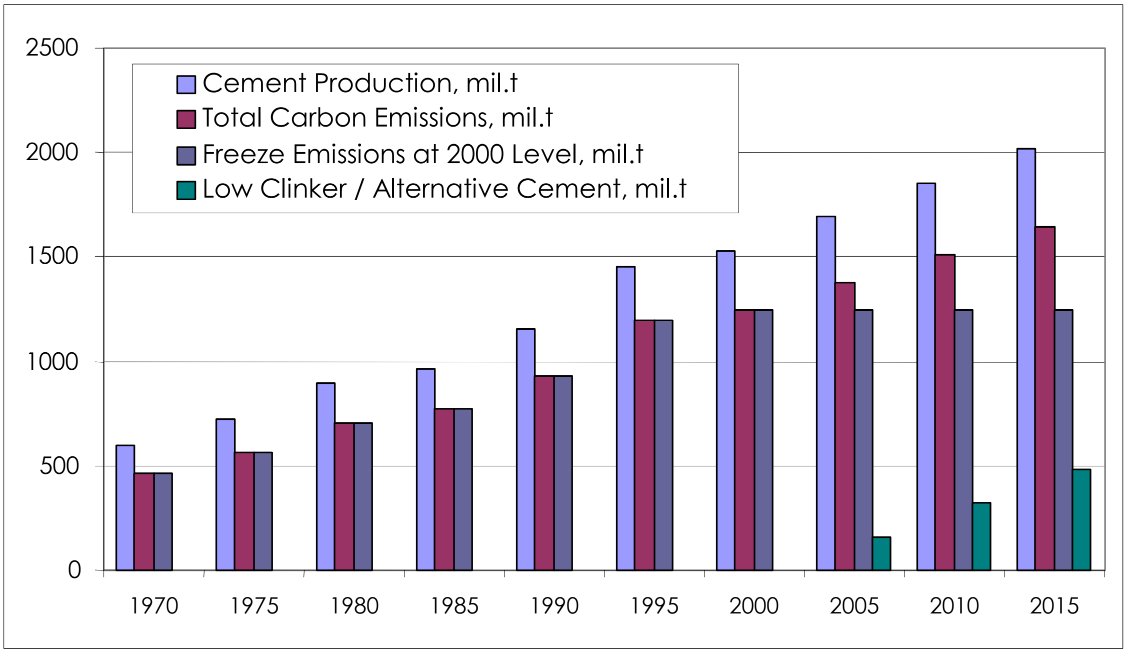

**FIGURE 4**. Prediction of emissions and market share of low-clinker cements.

At the same time, the extensive updating of existing facilities for the manufacture of clinker (which comprises an essential part of the inferior cements) consumes the bulk of capital investment and yields only a slow return[43]. Therefore, the expansion of an existing cement plant requires a proportionally high rate of investment. The major part of this investment is associated with the installation of heavy equipment and construction. However, in the case of HVMA cement, new investments are required only to upgrade the grinding unit. This may increase production capacity by 40–50% with consequent increase in profit, but without any additional increase of clinker output (Table 3).

Since HVMA cement uses about 30–50% less clinker than inferior cements, it creates less ecological damage. In this way HVMA cement contributes to the reduction of $CO_2$ and other emissions at source; IBPW materials that would be otherwise transported to landfill sites are used economically.

Considering the superior level of properties of HPC-based materials, the most important ecological and economical aspects contributing to the sustainable development are the following[42]:

- Reduced cross-sections of structures for concrete raw materials, structural steel, and energy saving

**TABLE 3**
**Expanding an Existing Cement Plant: Case of HVMA Cement[43]**

| Performance Parameters | Existing Plant | +20% Scenario | +40% Scenario |
|---|---|---|---|
| Mineral Additive Content, *%* | 30 | 50 | 70 |
| Capacity, *mil. tons per year* | 1.0 | 1.4 | 2.3 |
| Required Investments, *mil. $* | — | 4.3 | 13.9 |
| Unit Price, *$/ton* | 45 | 45 | 45 |
| Income, *mil. $* | 45.0 | 63.0 | 103.5 |
| Production Cost, *mil. $* | 39.4 | 53.1 | 83.5 |
| Gross Profit, *mil. $* | 5.6 | 9.9 | 20.0 |
| Extra Gross Profit, *mil. $* | — | 4.3 | 14.4 |
| Extra Net Profit, *mil. $* | — | 3.4 | 11.5 |
| Pay Back Period (10% interest rate), *year* | — | 1.4 | 1.3 |

- Reduced total weight of structures and foundations, reduced total energy consumption, less formwork, reduced maintenance costs and equipment loading, increased speed of erection
- Significantly increased lifetime of structures and reduced cost of repairing work because of high durability
- Replacement of generally used structural steel, polymers, and natural stone by ecologically friendly concrete
- Utilization of IBPW in HP/HVMA cement and reduction of energy consumption per unit of the cement produced
- Decreased amount of land spoiled by IBPW

## CONCLUDING REMARKS

1. HVMA cement uses less clinker than existing cements and so meets the challenges of a sustainable society; it increases bulk production and conserves energy. HVMA cement contributes to the reduction of $CO_2$ and other emissions at source. Due to its better eco-compatibility, the market share of HVMA cements will increase in the future.
2. Because of its higher strength and durability, the production and application of HP/HVMA blended cement helps to reduce the environmental degradation associated with construction activities.
3. Because of its ecological and economical advantages an ECO-prefix can be suggested for HP/HVMA cement: ECO-cement.

## ACKNOWLEDGMENT

The author would like to acknowledge the contributions of reviewers and colleagues at LAU which were useful to improve this article.
